# Effect of fat extraction methods on the fatty acids composition of bovine milk using gas chromatography

**DOI:** 10.1002/fsn3.2252

**Published:** 2021-05-04

**Authors:** Asmaa H. M. Moneeb, Ahmed R. A. Hammam, Abdelfatah K. A. Ahmed, Mahmoud E. Ahmed, Khalid A. Alsaleem

**Affiliations:** ^1^ Department of Dairy Science Faculty of Agriculture Assiut University Assiut Egypt; ^2^ Dairy and Food Science Department South Dakota State University Brookings SD USA; ^3^ Department of Food Science and Technology Faculty of Agriculture Assiut University Assiut Egypt; ^4^ Department of Food Science and Human Nutrition College of Agriculture and Veterinary Medicine Qassim University Buraydah Saudi Arabia

**Keywords:** fat extraction, gas chromatography, method validation, milk fat, milk fat yield, milk fatty acid composition

## Abstract

Milk fat is a complex natural fat and contains around 400 fatty acids. The objectives of this study were to extract fat from bovine milk using two different methods, including Bligh and Dyer and Mojonnier, and to determine the fatty acid content in the extracted fats using gas chromatography (GC). No differences (*p* > .05) were detected in the fat content and fatty acids content as a percentage of total fat (FA%TF) extracted using both methods. No differences (*p* > .05) were detected in some saturated fatty acids (SFAs) and unsaturated fatty acids (USFAs) extracted from both methods, such as C11:0 (undecylic acid), C16:0 (palmitic acid), C18:0 (stearic acid), C14:1 (myristoleic acid), and C16:1 (palmitoleic acid). However, the majority of SFAs were different (*p* < .05) in Mojonnier method as compared to Bligh and Dyer method and vice versa for USFAs. The short (6.54% vs. 5.95%) and medium (21.86% vs. 20.73%) chains FAs determined by GC were high in Mojonnier fat as compared to Bligh and Dyer fat, while the long‐chain FAs were higher in the last (66.61%) relative to Mojonnier fat (65.51%). This study found that Mojonneir method has resulted in fewer errors. In contrast, the Bligh and Dyer extraction method has more experimental error, which led to decreasing the total fat, as well as was not able to detect C9:0.

## Graphical abstract

This study found that Mojonneir method for fat extraction has resulted in fewer errors. In contrast, the Bligh and Dyer extraction method has more experimental error, which led to decreasing the total fat, as well as was not able to detect C9:0.

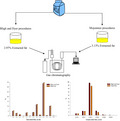

## 1 INTRODUCTION

Milk has nutritional and physiological characteristics that made it attractive (Miller et al., 2006). Bovine milk consists of protein (3%–4%), fat (3%–5%), lactose (4%–5%), and water (85%–87%). The composition of milk can be different based on the feeding, lactation period, and breeding (Lindmark‐Månsson et al., 2003; Walstra et al., 1999; Walstra & Jenness, 1984). Milk fat (MF) is a versatile ingredient because of its nutritional value, functionality, and flavor (Alsaleem, 2019). The fat in bovine milk exists as globules in water. Fat globules are coated with protein, phospholipids, cholesterol, and other components that form the globule membrane, which in turn keeps the emulsification characteristics of fat. The size of fat globules has a range of <1–10 µm (Jensen, 2002). Determination of fat became an important analysis in the dairy industry due to the significant role of MF. MF is presented in the form of triacylglycerols (TAGs), diacylglycerols, monoacylglycerols, cholesterol, free fatty acids, and phospholipids, accounting for 97.5%, 0.36%, 0.02%, 0.31%, 0.02%, and 0.6% of the total fat, respectively (Fox et al., 2015; Jensen, 2002; Gordon et al., 2013). TAGs are a main molecular form of MF containing 3 fatty acids (FAs) esterified to a glycerol backbone. In contrast, diacylglycerols, monoacylglycerols, free fatty acids, polar lipids and sterols, and trace amounts of vitamins are presented in MF as fat‐soluble material. More than 400 individual FAs have been identified in MF (Kontkanen et al., 2011), from which approximately fifteen FAs made up around 90% of the total MF (Lucey et al., 2017). FAs are characterized by the number of carbons and degree of saturation.

There are two main sources of FAs: the first is coming from the food, and the second is the activity of microbes in the cow's rumen (Parodi, 2004). The FAs are synthesized in the mammary gland with even carbon numbers (4–16) with around 60 molar and 45% weight basis (Fox, 2002). The synthesis in the mammary gland produces fatty acids from 4:0 to 14:0 with producing approximately 50% of 16:0 from acetate and β‐hydroxybutyrate. The fermentation of feed in the rumen of cows is generating acetate and butyric acids. During the absorption in the epithelium of rumen, butyric acid converts to β‐hydroxybutyrate. Additionally, MF has fatty acids with odd carbon numbers, including pentadecanoic acid (15:0) and heptadecanoic acid (17:0) (Mansson, 2008). Those two FAs are generated by the microflora in the cow's rumen (German & Dillard, 2006). The dietary lipids and lipolysis of tissue triacylglycerols have a significant role in synthesizing the rest of 16:0 and long‐chain FAs (Parodi, 2004). To produce the monosaturated acids, the 18:0 (medium‐ and long‐chain FAs) might desaturate in the mammary gland (Mansson, 2008).

Every FA has a specific position on the triacylglycerol molecule to esterifies at (MacGibbon & Taylor, 2006). For example, butyric acid (4:0) and caproic acid (6:0) are short‐chain acids and their esterification preference is *sn*‐3, while 8:0 to 16:0 (medium FAs) prefer to esterify in the position of *sn*‐1 and *sn*‐2. The 18:0 (stearic acid) is positioned at *sn*‐1 and 18:1 placed at the position of *sn*‐1 or *sn*‐3 (Mansson, 2008). The triacylglycerol is lipolyzed first in the human mouth by lingual lipase and second in the stomach by lingual lipase and gastric lipase (Parodi, 2004). When the triacylglycerol is lipolyzed, FAs in the position of sn‐3 are hydrolyzed to produce short FAs (4:0 to 10:0) to get through the stomach wall. Then, they pass to the portal vein, transport to the liver for oxidization. The stomach can digest approximately 25%–40% of the triacylglycerols (Jensen, 2002).

Fatty acids can be separated into two major categories: saturated and unsaturated FAs. Saturated fatty acids (SFAs) contain no double bonds as other carbon atoms, and hydrogen atoms surround each carbon atom. These fatty acid molecules are joined in a zigzag chain as there is freedom rotation about the carbon atoms due to eh absence of double bonds, such as C4:0—butyric acid, C6:0—caproic acid, C8:0—caprylic acid, C10:0—capric acid, C12:0—lauric acid, C14:0—myristic acid, C15:0—pentadecylic acid, C16:0—palmitic acid, C17:0—margaric acid, and C18:0—stearic acid. Unsaturated fatty acids (USFAs) contain carbon to carbon double bonds. These FAs are further classified as monounsaturated (one double bond), or polyunsaturated (more than one double bond), including C16:1—palmitoleic acid, C18:1—oleic acid, C18:2—linoleic acid, and C18:3—α‐linolenic acid (Jensen, 2002).

Most MF is SFAs, which is approximately 70%, while USFAs represent around 30% (Mansson, 2008). Several studies have reported that SFAs provide different bioactive fatty acids, such as short‐chain fatty acids and other minor functional compounds (phospholipids and sphingolipids) which in turn have positive impacts on human health (Semih & Selin, 2014). However, consumption of SFAs frequently is resulting in severe health issues, including cardiovascular diseases, cancers, and obesity (Jiménez‐Colmenero et al., 2001).

Since the importance of FAs and the concerns about the FAs in healthy foods (Küllenberg et al., 2012; Martínez‐Monteagudo et al., 2014; Merrill et al., 1997), then extraction and quantification of the FAs content in foods are essential for consumer's health. Extraction of fat is required solvents to separate the fat, and then, solvents are evaporated. Different methods have been proposed and used to extract the MF, such as centrifugation (Feng et al., 2004), dry column (Maxwell et al., 1986), dichloromethane (CH_2_Cl_2_)—ethanol (Stefanov et al., 2010), densitometer (Badertscher et al., 2007), Mojonnier (Case et al., 1985; Hooi et al., 2004), and Bligh and Dyer method (Bligh & Dyer, 1959). Each method has different procedures and thereby different chemicals that affect the extracted fat and FAs content. As a result, variations in the fat content and FAs were reported. Few works have elaborated on comparison methods of fat extraction. Bligh and Dyer and Mojonnier methods have been recommended, especially when the MF is higher than 3.5% (Jensen, 2002). Therefore, the objectives of this study are to extract and quantify the fat content in milk using the most popular fat extractions (Bligh and Dyer and Mojonnier) and to determine the SFAs and USFAs contents in MF using gas chromatography (GC).

## 2 MATERIALS AND METHODS

### 2.1 Extraction of fat from milk

#### 2.1.1 Mojonnier method

Mojonnier method was used to determine the fat content in bovine milk (obtained from the commercial market). In a Mojonnier tube, 10.0 g of milk, 3.0 ml of ammonium hydroxide (Fisher Scientific, Fair Lawn, NJ), and 3–6 drops of phenolphthalein (BCCA Chemical Company, Arlington, TX) were added. Three extractions were performed to extract the majority of the fat. First extraction: 13, 25, and 25 ml of ethyl alcohol (Fisher Scientific, Fair Lawn, NJ), ethylene ether (Fisher Scientific, Geel, Belgium), and petroleum ether (Fisher Scientific, Geel, Belgium) were added, respectively. Then, centrifugation was done for 1 min. Afterward, the colorless portion was removed in preweighed aluminum plates and then dried at a plate heater at low temperatures to avoid burning the sample. Second extraction: The same chemicals were added including ethyl alcohol (C_2_H_5_OH), ethylene ether (C_4_H_10_O), and petroleum ether (C_6_H_14_) at a rate of 5, 15, and 15 ml, respectively. Subsequently, centrifugation was done followed by pouring the colorless portion. Third extraction: It was similar to the second one. The plates left for 2–3 hr for drying in the oven at 103°C, cooled, and weights were recorded subsequently. This experiment was tri replicated.

$$\%\;\text{Fat}=\frac{\text{Final}\;\text{plate}\;\text{weight}‐\text{initial}\;\text{plate}\;\text{weight}}{\text{Sample}\;\text{weight}\;\left(10\;g\right)}$$

#### 2.1.2 Bligh and Dyer method

The milk fat was extracted using the same procedures of Bligh and Dyer (Bligh & Dyer, 1959). A 50 ml centrifuge tube was utilized to weigh 5 g of bovine milk and 650 mg of distilled water. A 5 ml of chloroform (Fair Lawn, NJ 07410) and a 10 ml of methanol were added into the tubes and then vortex for 2 min at low speed to avoid emulsions that form at high speed. Afterward, 5 ml of chloroform and 5 ml of distilled water were also added and vortex for 30 s, and then, centrifugation (CR 4–12, Jouan centrifuge) was done for 20 min at 454 *g*. The top layer that consisted of water and chloroform was removed using a Pasteur pipette. The remaining was filtered in a preweighed glass scintillation vials Whatman paper #1 (Cat No 1001 125). After that, 5 ml of chloroform was used to rinse the filter. The extracted filtrate was then gently dried using air stream; then, dry air was used for evaporating the chloroform for 60–90 min to complete drying. Afterward, the weight of vials was recorded; then, the fat content was calculated in milk using the same formula as in Mojonnier method. Finally, the extracted samples were kept at 4°C for GC.

### 2.2 Determination of FAs using GC

Butylation and separation method of FAs by GC was adopted from Sukhija and Palmquist (1988). The butanol (Fair Lawn, NJ 07410) was added to each sample to concentrate 35 mg fat/ml. The diluted lipid was then transferred into a screw‐capped extraction tube (13 × 100 mm). Then, 25 µl of tridecanoic acid (an internal standard; C 13:1) was added to each of the extraction tubes and vortex for 2–4 s. Afterward, 50 µl of acetyl chloride (Fair Lawn, NJ 07410) was added precautiously during vortex at low speed. The acetyl chloride was added cautiously. The nitrogen was added into the tubes for 2–4 s, and then, the tubes were capped tightly and placed on the heating block at 60°C for 90 min.

**FIGURE 1:**
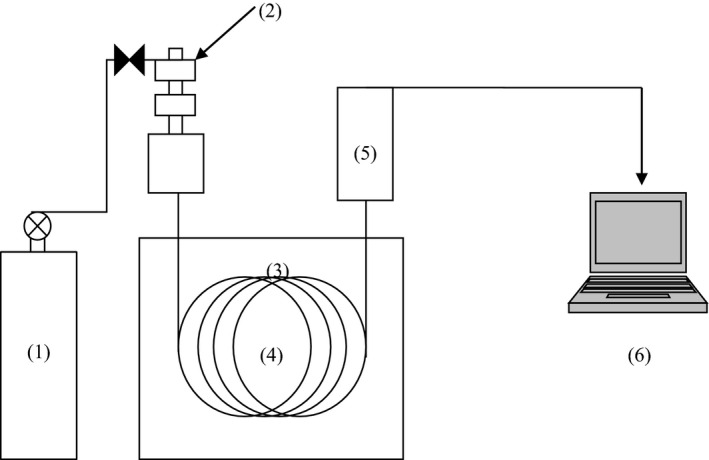
Gas chromatography (GC) system: (1) gas cylinder, (2) sample injection, (3) column oven, (4) column, (5) detector, and (6) data processing

The tubes were then cooled to room temperature, and 5 ml of 6% potassium carbonate (Fair Lawn, NJ 07410) solution was added and vortex for 30 s. Consequently, 1 ml of hexane (Fair Lawn, NJ 07410) was added and then vortex again for 30 s. After this, the tubes were centrifuged at 1,260 *g* at 4°C for 20 min. The lower layer (water and butanol) was then removed with a Pasteur pipette. Then, 5 ml of distilled water was added to the remaining hexane (containing the butyl esters of FAs) and vortex for 30 s. The same centrifugation conditions (1,260 *g* at 4°C for 20 min) were applied to centrifuge the tubes again, and then, the lower layer was discarded. This step was repeated two more times. At least 500 µl of the top layer (hexane and butyl esters of FAs) was pipette into GS vials using Pasteur pipettes. Transferring was done cautiously to transfer only the hexane layer with no mixed water. In all samples, FAs were measured using Hewlett–Packard 5890 gas–liquid chromatography equipped with automatic sampler 7673A, integrator 3392A, and FID detector (Figure 1).

### 2.3 Statistical analyses

Results were analyzed by R software (R x64‐3.3.3). All data were analyzed by ANOVA using a GLM for each variable to study the effect of each method on the FAs and fat content. The least significant difference (LSD) comparison test was used to determine significant differences between means at *p* < .05.

## 3 RESULTS

### 3.1 Milk fat

The fat content extracted from milk using Bligh and Dyer and Mojonnier methods and FAs as a percentage of total fat, SFAs, and USFAs contents are exemplified in Table 1. No significant difference (*p* > .05) was detected in the amount of fat extracted using Mojonnier (3.13 ± 0.07%) and Bligh and Dyer (2.97 ± 0.20%). Bligh and Dyer's method resulted in more variations in the amount of extracted fat. Mojonnier method is more efficient than Bligh and Dyer, and this was shown in the amount of extracted fat.

**TABLE 1:** The mean (*n* = 3 ± *SEM*) of milk fat extracted by Bligh and Dyer and Mojonnier methods, fatty acids as a percentage of total fat (FA%TF), saturated fatty acids (SFAs), and unsaturated fatty acids (USFAs) contents measured by gas chromatography (GC)

%	Bligh and Dyer	Mojonnier
Fat	2.97 ± 0.20	3.13 ± 0.07
Fatty acids as total fat	56.84 ± 2.55	53.04 ± 0.06
Saturated fatty acids	73.83 ± 0.05^b^	76.87 ± 0.05^a^
Unsaturated fatty acids	26.13 ± 0.05^a^	23.10 ± 0.05^b^

The percentage of FAs as a percentage of total fat (FA%TF) extracted from milk using Bligh and Dyer's method was slightly higher but not significant (*p* > .05) relative to Mojonnier method (Table 1). The FA%TF in milk was 56.84 ± 2.55 and 53.04 ± 0.06% using Bligh and Dyer and Mojonnier methods, respectively. However, the percentage of SFAs and USFAs determined using GS was significantly different (*p* < .05) between both methods. The SFAs was 73.83 ± 0.05% obtained from Bligh and Dyer method and 76.87 ± 0.05% resulted from Mojonnier method, while the USFAs was 26.13 ± 0.05 and 23.10 ± 0.05% in Bligh and Dyer and Mojonnier methods, respectively.

### 3.2 Fatty acids in milk fat

**FIGURE 2:**
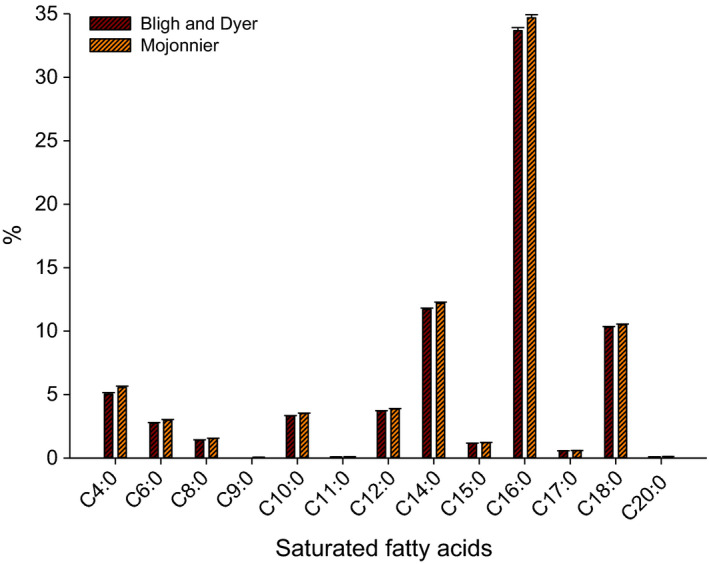
The saturated fatty acids (SFAs) in milk fat separated using Bligh and Dyer and Mojonnier methods and determined by gas chromatography (GC)

Table 2 is presented the fatty acid components of Bligh and Dyer and Mojonnier methods, retention time, and areas using GC. Eighteen FAs were detected in MF of each method using GC. Figure 2 is illustrated the SFAs in MF of Bligh and Dyer and Mojonnier methods determined by GC. The SFAs were 73.83 ± 0.05% obtained from Bligh and Dyer method and 76.87 ± 0.05% resulted from Mojonnier method. Table 2 is shown that the retention time of SFAs was similar in the extracted fat from both methods. However, the area of SFAs extracted from Mojonnier method was slightly higher than those produced from Bligh and Dyer method, which resulted in a higher percentage of those SFAs inMojonnier method. Thirteen SFAs were detected in milk fat, including C4:0—butyric acid, C6:0—caproic acid, C8:0—caprylic acid, C9:0—pelargonic acid, C10:0—capric acid, C11:0—undecylic acid, C12:0—lauric acid, C14:0—myristic acid, C15:0—pentadecylic acid, C16:0—palmitic acid, C17:0—margaric acid, C18:0—stearic acid, and C20:0—arachidic acid, which presented by 3.70%, 2.25%, 1.35%, 0.0%, 3.15%, 0.09%, 3.56%, 10.67%, 1.04%, 34.28%, 0.48%, 9.05%, and 1.46%, respectively, in Bligh and Dyer method and 4.11%, 2.43%, 1.51%, 0.06%, 3.35%, 0.10%, 3.76%, 11.15%, 1.10%, 35.49%, 0.50%, 9.27%, and 0.10%, respectively, in Mojonnier method (Table 2). The C9:0 (pelargonic acid) was not detected in the extracted fat of Bligh and Dyer method, while it presents by 0.06% in the fat of Mojonnier method (Figure 2).

**TABLE 2:** Calculation results for fatty acids components using gas chromatography (GC)

Fatty acid ID	Bligh and Dyer method			Mojonnier method		
	Time	Area, pA.s	Area, %	Time	Area, pA.s	Area, %
C4:0—butyric acid	14.14	61.40	3.70	14.12	67.78	4.11
C6:0—caproic acid	18.21	37.35	2.25	18.19	40.10	2.43
C8:0—caprylic acid	22.20	22.45	1.35	22.19	24.83	1.51
C9:0—pelargonic acid	0.0	0.0	0.0	24.42	0.98	0.06
C10:0—capric acid	26.60	52.07	3.15	26.58	55.27	3.35
C11:0—undecylic acid	29.46	1.42	0.09	29.44	1.58	0.10
C12:0—lauric acid	32.74	58.89	3.56	32.71	61.98	3.76
C14:0—myristic acid	43.42	176.59	10.67	43.37	183.77	11.15
C14:1—myristoleic acid	47.62	14.52	0.87	47.58	13.72	0.83
C15:0—pentadecylic acid	51.64	17.28	1.04	51.60	18.17	1.10
C16:0—palmitic acid	55.95	566.90	34.28	55.91	584.96	35.49
C16:1—palmitoleic acid	56.94	33.34	2.02	56.90	31.97	1.94
C17:0—margaric acid	59.67	7.90	0.48	59.63	8.19	0.50
C18:0—stearic acid	63.55	149.92	9.05	63.50	152.89	9.27
C18:1—oleic acid	64.22	298.28	18.01	64.16	270.51	16.41
C18:2—linoleic acid	66.44	37.28	2.25	66.39	26.52	1.61
C18:3—α‐linolenic acid	69.89	5.45	0.33	69.84	3.09	0.19
C20:0—arachidic acid	72.24	1.46	0.09	72.21	1.65	0.10
C20:3—dihomo‐α‐linolenic acid	77.99	1.66	0.10	0.0	0.0	0.0

Kaylegian and Lindsay (1995) have reported similar ranges for the SFAs in milk fat. They reported that C4:0—butyric acid, C6:0—caproic acid, C8:0—caprylic acid, C10:0—capric acid, and C12:0—lauric acid ranged from 2% to 5%, 1% to 5%, 1% to 3%, 2% to 4%, and 2% to 5%, respectively. Additionally, they found that C14:0—myristic acid, C15:0—pentadecylic acid, C16:0—palmitic acid, C17:0—margaric acid, and C18:0—stearic acid at the range of 8%–14%, 1%–2%, 22%–35%, 0.5%–1.5%, and 9%–14%, respectively. The results of our study were in the range of that reported by Kaylegian and Lindsay.

**FIGURE 3:**
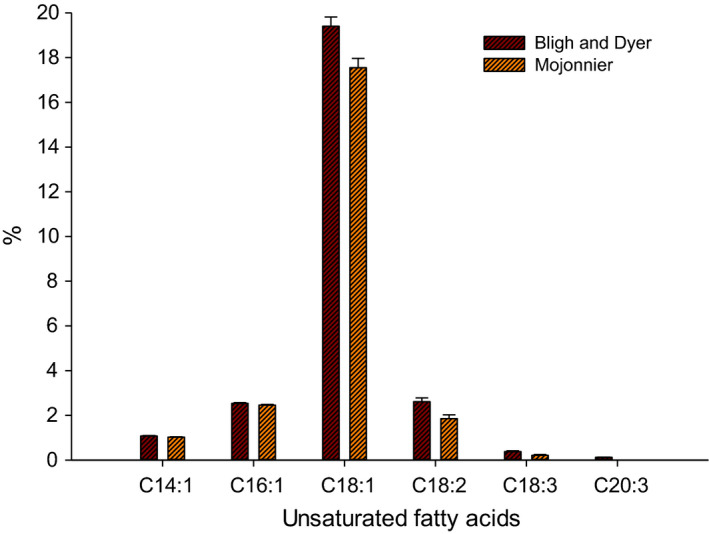
The unsaturated fatty acids (USFAs) in milk fat separated using Bligh and Dyer and Mojonnier methods and determined by gas chromatography (GC)

The USFAs in fat extracted from Bligh and Dyer and Mojonnier methods are shown in Figure 3. The total USFAs were 26.13 ± 0.05 and 23.10 ± 0.05% in Bligh and Dyer and Mojonnier fats, respectively. The retention time of USFAs was similar in the extracted fat from both methods as in SFAs. However, the area of USFAs extracted from Mojonnier method was slightly lower than those produced from Bligh and Dyer method, which led to a lower percentage of those USFAs in Mojonnier method. Six USFAs were detected in milk fat, including C14:1—myristoleic acid, C16:1—palmitoleic acid, C18:1—oleic acid, C18:2—linoleic acid, C18:3—α‐linolenic acid, and C20:3—dihomo‐α‐linolenic acid, which found by 0.87%, 2.02%, 18.01%, 2.25%, 0.33%, and 0.10%, respectively, in Bligh and Dyer method and 0.83%, 1.94%, 16.41%, 1.61%, 0.19%, and 0.0%, respectively, in Mojonnier method (Table 2). The C20:3 (dihomo‐α‐linolenic acid) was not detected in the extracted fat of Mojonnier method, while it presents by 0.1% in the fat of Bligh and Dyer method (Figure 3).

Kaylegian and Lindsay (1995) also reported similar ranges for the USFAs in milk fat. They reported that C16:1—palmitoleic acid, C18:1—oleic acid, C18:2—linoleic acid, and C18:3—α‐linolenic acid were ranged from 1% to 3%, 20% to 30%, 1% to 3%, and 0.5% to 2% in milk fat, respectively. The results of our study were in the range of that reported by Kaylegian and Lindsay.

Short‐chain FAs, such as 4:0 and 6:0, were lower in Bligh and Dyer method (5.95%) as compared to Mojonnier method (6.54%). In addition, the medium‐chain FAs (8:0 to 15:0) determined by GS were 20.73% in Bligh and Dyer fat as compared to 21.86% in Mojonnier fat. However, the long‐chains FAs were higher in Bligh and Dyer method (66.61%) as compared to Mojonnier method (65.51%)

## 4 DISCUSSION

### 4.1 Milk fat

The variations in fat and fatty acids content produced from both methods can be obtained from the loss of some lipid that occurred unwittingly with the top layer during extraction (Table 1). The loss of lipid contributed to reducing the percentage of extracted fat from milk samples. It has been found that the Bligh and Dyer method is not accurate in measuring the fat content, especially with increasing the fat content (Arnould et al., 1995; Oftedal et al., 2014). This is also another reason for having less fat content in Bligh and Dyer method as compared to Mojonnier method. However, McCarthy reported that the percentage of fat in bovine milk was 3.25%, which is similar to Mojonnier extraction method (McCarthy et al., 2017).

It has been reported that SFAs and USFAs ranged from 67.1% to 74.4% and 24.2% to 29.2% in bovine milk, respectively (Mansson, 2008). The SFA and USFAs contents determined by Bligh and Dyer and Mojonnier methods in our study were similar and in the range of Mansson's study. However, Bligh and Dyer's method showed more USFAs and fewer SFAs as compared to Mojonnier method and this can be due to the loss of some fat in Bligh and Dyer method during fat extraction plus the sensitivity of this method with the high‐fat content products which caused the variations (Arnould et al., 1995; Oftedal et al., 2014).

### 4.2 Fatty acids in milk fat

As shown in Figure 2, The individual SFAs determined in our study fall in the range reported in Mansson's study (Mansson, 2008). The dominant FAs in the SFAs is palmitic acid (C16:0) which represented approximately 50% of SFAs and around 33.67 ± 0.21% and 34.69 ± 0.12% of total FAs in Bligh and Dyer fat and Mojonnier fat, respectively, and this was similar to other studies (Mansson, 2008). Another study also reported that C16:0 was found in cow's lipids milk with 32.12 ± 1.68% (Castro‐Gómez et al., 2014). There were differences (*p* < .05) in the majority of SFAs of fat extracted from Bligh and Dyer and Mojonnier methods. The SFAs extracted from Mojonnier method, such as C4:0 (butyric acid), C6:0 (caproic acid), C8:0 (caprylic acid), C10:0 (capric acid), C12:0 (lauric acid), C14:0 (myristic acid), C15:0 (pentadecylic acid), C17:0 (margaric acid), and C20:0 (arachidic acid), were higher (*p* < .05) as compared to Bligh and Dyer method. However, no significant difference (*p* > .05) was detected in some SFAs extracted from both methods, such as C11:0 (undecylic acid), C16:0 (palmitic acid), C18:0 (stearic acid), and in somehow Mojonnier fat presented approximately 0.06% of C9:0 (pelargonic acid). In contrast, Bligh and Dyer's fat did not present this acid by GC. The differences in SFAs could have resulted from the Bligh and Dyer method's inaccuracy that led to not detecting the pelargonic acid in the extracted fat from this method (Arnould et al., 1995; Oftedal et al., 2014).

The USFAs determined in the fat of both methods were similar and fall in the range of Mansson's study. No significant difference (*p* > .05) was detected in the C14:1 (myristoleic acid) and C16:1 (palmitoleic acid) of FAs of both methods. The average of myristoleic acid (C14:1) and palmitoleic acid (C16:1) in both methods was 1.0%, and 2.5%, respectively, which is similar to Mansson's study. However, our study showed a little higher percentage of palmitoleic acid and this due to the differences in milk compositions and feeding diet. On the other hand, significant differences (*p* < .05) were detected in the USFAs resulted from both methods, including C18:1 (oleic acid), C18:2 (linoleic acid), and C18:3 (α‐linolenic acid). Those USFAs were relatively higher (19.40 ± 0.07, 2.61 ± 0.005, 0.38 ± 0.00, respectively) in Bligh and Dyer method as compared to Mojonnier method (17.55 ± 0.15, 1.85 ± 0.01, 0.21 ± 0.0, respectively). Additionally, the fatty acid C20:3 (dihomo‐α‐linolenic acid) was detected in Bligh and Dyer fat and was not found in Mojonnier fat which could be due to the efficiency of Mojonnier solvents used to extract MF. A similar trend has been found in the USFA of MF (Castro‐Gómez et al., 2014; Mansson, 2008; Sukhija & Palmquist, 1988).

## 5 CONCLUSIONS

This work aimed to extract fat from bovine milk using Bligh and Dyer and Mojonnier methods to determine the FAs content in the extracted fats using GC. No differences (*p* > .05) were detected in the fat content and FA%TF extracted using both methods. Additionally, no differences (*p* > .05) were detected in SFAs and USFAs extracted from both methods, such as C11:0 (undecylic acid), C16:0 (palmitic acid), C18:0 (stearic acid), C14:1 (myristoleic acid), and C16:1 (palmitoleic acid). However, the SFAs were higher (*p* < .05) in Mojonnier method relative to Bligh and Dyer method while USFAs were high in the remaining as compared to Mojonnier method. Mojonnier method can be a suitable method to extract the MF and determine the FAs using GC.

## CONFLICT OF INTEREST

The authors have no conflict of interest to declare.

## Data Availability

Research data are not shared.
